# Microglia and the urokinase plasminogen activator receptor/uPA system in innate brain inflammation

**DOI:** 10.1002/glia.20892

**Published:** 2009-05-20

**Authors:** Orla Cunningham, Suzanne Campion, V Hugh Perry, Carol Murray, Nicolai Sidenius, Fabian Docagne, Colm Cunningham

**Affiliations:** 1 FIRC Institute of Molecular Oncology20139 Milan, Italy; 2 INSERM U919 “Serine Proteases and Pathophysiology of the Neurovascular Unit,” UMR CINAPS—CNRS/CEA/UCBN/Université Paris DescartesCaen, France; 3 CNS Inflammation Group, School of Biological SciencesBassett Crescent East, Southampton SO16 7PX, United Kingdom; 4 Trinity College Institute of Neuroscience, TCDDublin 2, Ireland

**Keywords:** chronic neurodegeneration, neuroinflammation, plasminogen, proteolysis, activation

## Abstract

The urokinase plasminogen activator (uPA) receptor (uPAR) is a GPI-linked cell surface protein that facilitates focused plasmin proteolytic activity at the cell surface. uPAR has been detected in macrophages infiltrating the central nervous system (CNS) and soluble uPAR has been detected in the cerebrospinal fluid during a number of CNS pathologies. However, its expression by resident microglial cells *in vivo* remains uncertain. In this work, we aimed to elucidate the murine CNS expression of uPAR and uPA as well as that of tissue plasminogen activator and plasminogen activator inhibitor 1 (PAI-1) during insults generating distinct and well-characterized inflammatory responses; acute intracerebral lipopolysaccharide (LPS), acute kainate-induced neurodegeneration, and chronic neurodegeneration induced by prion disease inoculation. All three insults induced marked expression of uPAR at both mRNA and protein level compared to controls (naïve, saline, or control inoculum-injected). uPAR expression was microglial in all cases. Conversely, uPA transcription and activity was only markedly increased during chronic neurodegeneration. Dissociation of uPA and uPAR levels in acute challenges is suggestive of additional proteolysis-independent roles for uPAR. PAI-1 was most highly expressed upon LPS challenge, whereas tissue plasminogen activator mRNA was constitutively present and less responsive to all insults studied. These data are novel and suggest much wider involvement of the uPAR/uPA system in CNS function and pathology than previously supposed. © 2009 Wiley-Liss, Inc.

## INTRODUCTION

The urokinase plasminogen activator (uPA) receptor (uPAR) is a GPI-linked cell-surface protein that is best characterized as a region-specific focus for uPA protease activity at the cell surface. Binding of uPA to uPAR greatly accelerates the cleavage of plasminogen to active plasmin, and the receptor acts to concentrate this activation at discrete membrane locations. This is believed to be a key event in cell adhesion and migration and has been extensively researched in cancer metastases [see (Blasi and Carmeliet,^2002^) for review]. Monocytes/macrophages are important migratory cells and uPAR has been shown to be expressed by cells of the monocyte lineage (Min et al.,^1992^; Vassalli et al.,^1992^) and to be inducible by cytokines, in particular, transforming growth factor β1 (TGFβ1) (Lund et al.,^1991^). Under normal conditions, uPAR expression is negligible and uPAR knockout mice were initially found to be phenotypically normal but further study revealed that, under challenge, they displayed defective recruitment and migration of neutrophils and lymphocytes (Gyetko et al.,^2000^,^2004^). Low-level expression of uPAR has been shown in cultured microglial cells, and this expression was increased upon lipopolysaccharide (LPS) stimulation; however, uPAR could not be detected in microglia immediately *ex vivo* (Washington et al.,^1996^). Soluble uPAR (suPAR) is elevated in the cerebrospinal fluid (CSF) of patients with HIV dementia (Cinque et al.,^2004^), and it seems likely that this release of suPAR into the CSF is directly related to increased central nervous system (CNS) inflammation: uPAR has been described in macrophages/microglia within the CNS in traumatic brain injury, HIV dementia, multiple sclerosis, cerebral malaria, Creutzfeldt–Jakob disease (CJD), and Alzheimer's disease (Beschorner et al.,^2000^; Cinque et al.,^2004^; Deininger et al.,^2002^; Fauser et al.,^2000^; Gveric et al.,^2001^; Sidenius et al.,^2004^; Walker et al.,^2002^). However, clear direct evidence for resident (i.e. noninfiltrating) microglial expression of uPAR is still lacking, and its expression in different CNS insults has not been studied in any systematic way.

It has been recognized for some time that tissue plasminogen activator (tPA) is expressed and is constitutively active in the normal CNS (Sappino et al.,^1993^), in which it is proposed to have roles in synaptic plasticity, long-term potentiation, and neuronal migration [see Melchor and Strickland (2005) for review]. In addition, tPA activity is decreased in models of Alzheimer's disease, and this is thought to occur through increased expression of PAI-1 (Cacquevel et al.,^2007^; Melchor et al.,^2003^). Conversely, tPA activity is increased upon intracerebral injection of kainic acid, and this plasmin activity has a key role in the resulting neurotoxicity (Tsirka et al.,^1995^,^1997^). Increased tPA activity in models of prion disease have lead to the suggestion that frustrated attempts by plasmin to proteolytically cleave the deposited form of the prion protein result in bystander damage to neuronal elements during this disease (Ellis et al.,^2002^; Maissen et al.,^2001^). However, studies with tPA−/− and plasminogen−/− mice have yielded contradictory results (Salmona et al.,^2005^; Xanthopoulos et al.,^2005^). It is clear that tPA has multiple roles in both physiological and pathological situations in the brain. Conversely, uPA has generally been described as being absent from the normal CNS (Sappino et al.,^1993^). However, uPA is known to be stress responsive and is robustly induced during inflammation (Gabay and Kushner,^1999^). uPA is expressed in the inflamed CNS in a model of experimental allergic encephalopathy (East et al.,^2005^) and in cerebral ischemia in rodents and in humans (Rosenberg et al.,^1996^).

Because it is clear that there is complimentarity and redundancy in the plasmin cascade (Carmeliet et al.,^1994^), it is important to study, in detail, the expression of constituents of this system *in vivo* in addition to deleting selected genes. We have studied the expression of uPAR and uPA, as well as tPA and PAI-1, in the CNS in a number of insults that generate distinct and well-characterized inflammatory responses: acute nondegenerative inflammation induced by LPS (Andersson et al.,^1992^), acute neurodegeneration-associated inflammation induced by kainic acid (Andersson et al.,1991a,b), and chronic neurodegeneration-associated inflammation induced during prion disease (Betmouni et al.,^1996^; Cunningham et al.,^2002^; Walsh et al.,^2001^). Together, these insults generate a range of different innate inflammatory milieu in which uPAR and other components of the plasmin cascade may be expressed in the brain. These different insults induced alterations in the expression/activity of tPA and PAI-1 that were consistent with previous observations, but also revealed novel patterns of uPA and uPAR expression that suggest wider involvement of this system in the CNS than previously supposed.

## EXPERIMENTAL

### Animals and Stereotaxic Surgery

Female C57BL/6 mice were obtained from Harlan (Bicester, UK), housed in groups of five or six under standard light and temperature regimes, and fed with pelleted food and water *ad libitum*. For LPS challenges, animals were anesthetized with intraperitoneal avertin, positioned in a stereotaxic frame, and injected with 2.5 μg LPS (*Salmonella equine abortus*, Sigma, Poole, UK) in 1 μL, via a pulled glass microcapillary tube (coordinates from bregma: anterior–posterior −2.0 mm, lateral −1.6 mm, and depth −1.5 mm). For kainate challenges, 1 nmol of kainic acid (Sigma, Dorset, UK) was injected by exactly the same method as used for LPS. For inoculation with prion disease, animals were anesthetized in the same manner, and 1 μL of a 10% w/v ME7-infected C57BL/6 brain homogenate, made in phosphate-buffered saline (PBS), was injected bilaterally into the dorsal hippocampus (same coordinates) via a 10-μL Hamilton syringe. Control animals were injected with a 10% w/v normal brain homogenate (NBH) in PBS, derived from a naive C57BL/6 mouse. In addition, we made saline challenges and collected tissue from naive mice, both young adult and 8–9 months (age-matched to late stage ME7 and NBH animals). All procedures were carried out in accordance with UK Home Office license.

### Tissue Preparation

Animals challenged intracerebrally with LPS were terminally anesthetized at 6, 8, or 72 h post-LPS and examined for evidence of cell infiltration/inflammatory activation, because cytokine expression/microglial activation occurs significantly earlier (6/8 h) than macrophage and neutrophil infiltration (Andersson et al.,^1992^). Kainate-injected animals were euthanised at 24 h or 72 h post-injection. Neurodegeneration begins synchronously upon kainate challenge but significant macrophage infiltration occurs at 48 h post-kainate (Andersson et al.,1991a) and at 72 h most of the CA1 neurons of the hippocampus show clear morphological signs of nuclear condensation. Animals inoculated with ME7 prion disease were euthanized at 12, 15, 18, 20, and 23 weeks postinoculation for all mRNA studies, with NBH animals taken at 12 and 23 weeks for comparison. For protein expression studies in the ME7 model, this range was narrowed to 13, 18, and 21 weeks postinoculation to reduce animal usage. These times represent points in disease displaying hippocampal synaptic loss, hippocampal neuronal cell soma loss, and terminal disease, respectively (Cunningham et al.,2003a). Tissues were homogenized in PBS with Complete™ protease inhibitor cocktail (Roche) and centrifuged at 12,000 rpm for 10 min, and the supernatant was harvested (S1, representing soluble proteins). The pellet was then solubilized by incubation with1% triton-X100 for 1 h at 4°C and recentrifuged at 12,000 rpm for 10 min (P1, representing membrane-associated proteins).

All animals used for RNA extraction were terminally anesthetized using sodium pentobarbital, transcardially perfused with heparinized saline, and the brains removed (*n* = 5 for each animal group). Thick coronal sections (∼2 mm) were taken at ∼−1.0 to −3.0 mm from Bregma, and the hippocampus and dorsal thalamus were quickly removed, immediately frozen in liquid nitrogen, and stored at −80°C.

Animal groups for immunohistochemistry were terminally anesthetized and transcardially perfused with heparinized saline and fixed in 10% formalin solution (*n* = 3 for ME7, LPS, KA). Additional groups were perfused with freshly prepared paraformaldehyde-lysine-periodate fixative (PLP; 2% paraformaldehyde, 0.05% glutaraldehyde, 70 mM lysine, and 10 mM sodium periodate) to preserve the cell-surface markers uPAR and F4/80 (*n* = 3 for ME7, LPS, KA).

### RNA Extraction

Total RNA was extracted from brain samples using Qiagen RNeasy mini columns (Qiagen, Crawley, UK) according to the manufacturer's instructions. Contaminating genomic DNA was degraded during extraction with Qiagen *DNase1* enzyme. The typical yield for brain tissue was ∼5 μg per extraction. RNA was stored at −80°C until cDNA synthesis and PCR assay. Group sizes for mRNA extraction were as follows: NBH (*n* = 6), naïve, aged and 18-week ME7 (*n* = 4), 23-week ME7 (*n* = 5), and all others (*n* = 3).

### Taqman RT-PCR Assay

All equipment and reagents were supplied by Applied Biosystems (Warrington, UK) unless otherwise stated. Assays for PAI-1 and tPA were designed using the published sequences for these genes, applied to Primer Express software. Primer and probe sequences for uPA and uPAR were kindly supplied by Prof. Dylan Edwards. The sequences are shown in Table 1. Where possible, probes were designed to cross an intron to ensure that they were cDNA specific. Table 1 lists the sequences for primers and probes for each assay. All primer pairs were checked for specificity by standard RT-PCR using Promega PCR reagents (Southampton, UK) followed by gel electrophoresis. Each primer pair produced a discrete band of the expected amplicon size (not shown).

**TABLE 1:** Sequences of Primers and Probes Used in Quantitative TaqMan Analyses

Target	Oligonucleotide	Sequence	Amplicon size (bp)
tPA	Forward primer	5′-GGCCTGGCACGACACAAT-3′	66
	Reverse primer	5′-CATCACATGGCACCAAGGTC-3′	
	Probe	5′-ATTGTCGGAATCCAGATGGTGATGCC-3′	
PAI-1	Forward primer	5′-GGGACACCCTCAGCATGTTC-3′	69
	Reverse primer	5′-TGTTGGTGAGGGCGGAGA-3′	
	Probe	5′-TCGCTGCACCCTTTGAGAAAGATGT-3′	
uPA	Forward primer	5′-GAAACCCTACAATGCCCACAGA-3′	127
	Reverse primer	5′-GACAAACTGCCTTAGGCCAATC-3′	
	Probe	5′-CACAATTACTGCAGGAACCCTGACAAC-3′	
uPAR	Forward primer	5′-TGCAATGCCGCTATCCTACA-3′	116
	Reverse primer	5′-TGGGCATCCGGGAAGACT-3′	
	Probe	5′-CCCTCCAGAGCACAGAAAGGAGCTTGAA-3′	

For Taqman PCR, cDNA was generated from total RNA using Taqman Gold RT reagents. Two hundred nanograms of total RNA were reverse transcribed in a 10-μL reaction volume. One microliter of the RT reaction (equivalent to 20-ng RNA) was subsequently used for the PCR and performed as previously described (Cunningham et al.,^2005^). A standard curve was constructed from total RNA isolated from mouse brain tissue after intracerebral challenge with 2.5 μg LPS, which is known to upregulate all target transcripts of interest in this study. This standard curve was constructed using a higher concentration of RNA in the reverse transcriptase reaction than for samples for analysis to ensure that transcription in all animal groups would fall within the range of the standard curve constructed. Serial 1 in five dilutions of the cDNA synthesized from brains of LPS-injected mice were made and a curve plotted of the *C*_t_ value (the cycle number at which the transcribed gene crosses the threshold of detection) versus the log of the concentration (assigned an arbitrary value since the absolute concentration of cytokine transcripts is not known). Thus, the standard curve generates arbitrary values for concentration. These data were then normalized for GAPDH concentration in each sample.

### Western Immunoblotting

**Fig. 1:**
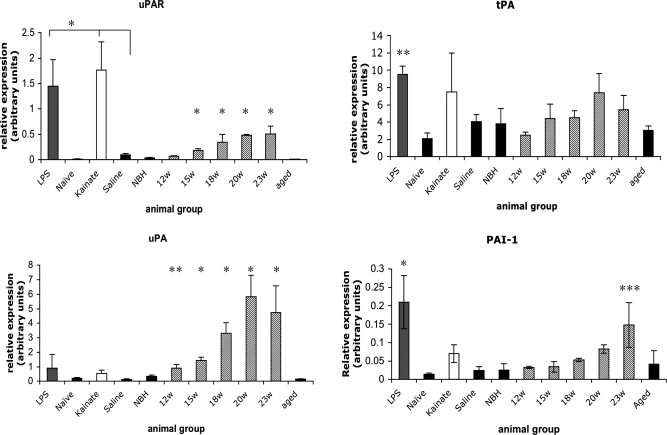
Quantitative PCR analysis of gene expression changes in uPAR, uPA, tPA, and PAI-1. Total RNA was isolated from hippocampus at various time points (LPS: 24 h, KA: 48 h, Saline, 24 h, and ME7 as described on *x*-axis). Synthesized cDNA was analyzed using TAQMAN PCR using specific primers and probes designed from published sequences for these genes. Statistical significance was determined by ANOVA with Bonferroni *post hoc* tests and *P* values are denoted by * (*P* < 0.001), **(*P* < 0.01), and ***(*P* < 0.05). Group sizes for mRNA extraction were as follows: NBH (*n* = 6), nave, aged and 18-week ME7 (*n* = 4), 23-week ME7 (*n* = 5), and all others (*n* = 3).

Pellet-enriched fractions were prepared as described for P1 (see tissue preparation) from LPS, kainate, and ME7 animals and analyzed for uPAR protein expression by western blot. Briefly, 25 μg of each lysate was loaded and run under reducing conditions on a 10% SDS–PAGE gel. Samples were transferred to PVDF, blocked in 5% nonfat milk in PBS-T (PBS and 0.2% Tween-20) for 2 h, and probed overnight with a polyclonal anti-mouse uPAR antibody (anti-smuPAR) at 1 μg/mL. The antibodies against uPAR, both polyclonal and monoclonal, were produced using recombinant smuPAR expressed in Schneider S2 cells using the Drosophila Expression System Kit (Invitrogen) as antigen. The protein was purified from the conditioned media of stably transfected S2 cells using Ni-NTA beads (Qiagen) followed by gel filtration. Rabbits and uPAR^−/−^ mice were immunized with recombinant smuPAR, and the IgG fraction of rabbit serum and hybridoma conditioned media were subsequently affinity purified on a column containing immobilized smuPAR (Sidenius, unpublished work). Detection was achieved with a 1:5,000 dilution of anti-rabbit-HRP or anti-mouse-HRP (GE Healthcare) and development with ECL reagent (PIERCE). The appropriate uPAR detection and specificity of this antibody were verified by transfection of Chinese Hamster Ovary cells with murine uPAR (Supp. Info. Fig. 1) and by staining of kidney tissue from wild type and uPAR−/− mice (Tjwa et al.,^2009^).

### Plasminogen Activation Assay

Samples were analyzed for plasminogen activation activity in an indirect enzymatic assay in a 96-well format using the chromogenic plasmin substrate Chromozym PL (Roche) with output at 410 nm. Reactions were carried out in assay buffer (50 mM Tris–HCl, pH 7.4, 100 mM NaCl) containing a final concentration of 0.2 μM plasminogen (Sigma), 0.2 mM chromozym PL, and 50-μg protein extract. Where a distinction between tPA and uPA activity was desired, selective inhibitors of each of the plasminogen activators were used. For tPA inhibition, a commerical tPA inhibitor, tPA Stop (American Diagnostica, Stamford, CT), was included in the reaction mix at a final concentration of 1.5 μM. uPA was inhibited using amiloride (Sigma) at a final concentration of 0.2 mM. Although these inhibitors are not entirely specific, they do show good selectivity at the concentrations used: 0.2 mM amiloride significantly inhibits uPA while having a minimal effect on tPA activity while the Ki of tPA STOP is 0.035 μM for tPA and 3.4 μM for uPA (Harvey and Chintala,^2007^). Samples were allowed to run overnight at room temperature, and data were collected using a DYNEX MRX plate reader using Revelation software (Worthing, UK).

### Gel zymography for tPA and uPA Activity

Enzyme activities for tPA and uPA were assessed using gel zymography. A substrate solution of 8% w/v Marvel dry milk in PBS, containing CaCl_2_ (0.9 mM) and MgCl_2_ (1.0 mM), was prepared. A 10% SDS−PAGE gel was prepared with 400 μL of 8% milk solution per 20 mL of resolving gel. In addition, 133 μL of plasminogen (1.5 mg/mL; Sigma P-7397) was added to the resolving gel. Solubilized pellets and supernatants (15-μg protein per lane) were added to loading buffer without mercaptoethanol and were not boiled before loading on the gel. Gels were run at 25 mA per minigel. The gels were rinsed in 2.5% Triton X-100 and then incubated in 10 mM CaCl_2_, 50 mM Tris (pH 7.6) for 6 h at 37°C. After this time, the gel was rinsed again and stained in Coomassie blue for 1 h before destaining until areas of proteolysis became apparent as clear areas in the intense blue staining of the remaining casein protein throughout the gel.

### Immunohistochemistry for uPAR and F4/80

Immunohistochemistry was carried out for uPAR and F4/80. All biotinylated secondary antibodies were supplied by Vector laboratories (Peterborough, UK) and all staining was visualised using the ABC method using peroxidase as enzyme, 0.015% v/v hydrogen peroxide as substrate and diaminobenzidine as chromagen. Frozen paraformaldehyde-lysine-periodate-fixed brain sections were cut on a cryostat and stored at −20°C. On removal from the freezer sections were dried at 37°C for 30 min. Endogenous peroxidase was quenched using methanol containing 1 ml 30% H_2_O_2_ for 20 min. Sections were washed with PBS-Tween (0.1%) and blocked using both Vector Avidin-Biotin block and Mouse-On-Mouse (MOM) blocking reagents. After 1 h of blocking with MOM blocking diluent and 5 min with MOM working solution, the primary monoclonal antibody BR4.8 (Sidenius, unpublished) was applied at 2 μg/ml and left to incubate in a humidified chamber for 2 h. This antibody was then washed off and sections were washed overnight in PBS-Tween. Biotinylated horse anti-mouse antibody was prepared in MOM diluent and left at room temperature for 30 min. ABC incubation and DAB staining were then performed as normal. Sections for F4/80 staining were oven dried and washed as before and then blocked for 30 min with normal 10% rabbit serum before incubation with the F4/80 antibody (Serotec, Oxford, UK) for 1 h at room temperature. The sections were then washed and incubated with biotinylated rabbit anti-rat antibody for 45 min and continued through ABC incubation and DAB/peroxidase reaction as before.

### Statistical Analysis

Gene expression data were analyzed by comparing all “acute” groups (naïve, saline, LPS, and KA) or all “chronic” groups (NBH, 12 weeks, 15 weeks, 18 weeks, 20 weeks, 23 weeks, and age-matched naive) by one-way ANOVA followed by selected pair-wise comparisons by Bonferroni *post hoc* tests. Significance was accepted at the 95% confidence interval.

## RESULTS

### Transcriptional Regulation of PA Components during CNS Inflammation

After RNA isolation from brain homogenates of treated mice, cDNA was synthesized and examined for transcription of uPAR, uPA, tPA, and PAI-1.

Transcription of uPAR was markedly increased for all challenges compared to their relevant control groups (Fig. 1a). LPS induced a 41-fold increase with respect to naïve animals and a 15-fold increase with respect to saline-injected animals (*P* < 0.001). KA induced a 50-fold increase with respect to naïve and an 18-fold increase with respect to saline-injected animals (*P* < 0.001). Injection of saline alone increased uPAR expression with respect to naïve animals. This was not statistically significant when all acute groups were compared, but when compared in the absence of LPS and KA groups, this difference is readily apparent (*P* < 0.001).

To investigate expression in chronic inflammation, we compared ME7 prion-diseased animals with NBH controls. NBH animals at 23 weeks postinoculation showed low-uPAR levels, similar to both aged-matched and young naïve animals (not significantly different by ANOVA with Bonferroni *post hoc* test). In contrast, ME7 animals showed a clear and time-dependent increase in expression of uPAR with disease progression, reaching ∼15-fold elevation compared to NBH animals by 20 weeks postinoculation. This increase is apparent by 12 weeks and statistically significant by 15 weeks (ANOVA with Bonferroni *post hoc* *P* < 0.001).

The mRNA for the protease uPA shows only variable and limited induction by LPS at 24 h or kainate at 72 h (Fig. 1b), and these increases are not statistically significant at these time points (no main effect by one-way ANOVA, no *post hoc* tests performed). In ME7 animals, by contrast, uPA transcription was markedly induced, by ∼17-fold by 20 weeks postinoculation. This increase is once again time-dependent, with induction already apparent and statistically significant at 12 weeks (Bonferroni *post hoc* *P* < 0.01). There were no significant differences between saline-injected, NBH-injected, or young or older naïve mice.

tPA is constitutively expressed in the brain and mRNA expression levels accordingly appear high at baseline and are less inducible (Fig. 1c). LPS challenge induced a moderate increase in expression with respect to naïve (*P* < 0.001) and to saline-treated animals (*P* < 0.01), but saline also induced a modest, though nonsignificant increase with respect to naïve animals (*P* > 0.05). tPA expression was induced in KA-injected animals (*P* < 0.01 versus naïve animals), but the induction was highly variable, and this increase did not reach statistical significance with respect to saline-treated animals. There is also a trend toward a time-dependent increase in tPA expression during prion disease progression, but even at peak expression, levels are only increased approximately twofold with respect to NBH controls and this does not reach statistical significance.

PAI-1 expression (Fig. 1d) was markedly induced by LPS compared to levels of expression in saline-treated animals (*P* < 0.001). PAI-1 was also induced in KA-injected animals but only to a modest and highly variable degree (nonsignificant, *P* > 0.05). PAI-1 was clearly induced with the progression of prion disease. This increase did not become statistically significant until 18 weeks (*P* < 0.05) and by 23 weeks was elevated approximately sixfold.

### uPAR Protein Expression During CNS Inflammation

**Fig. 2:**
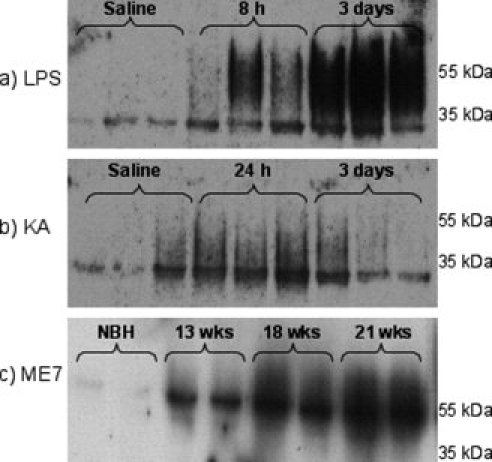
Western blot analysis of uPAR expression in hippocampal homogenates. Membrane fraction samples (25 μg protein) were separated by SDS–PAGE, transferred to PVDF, immunoblotted with a polyclonal antisoluble mouse uPAR antibody at 1 μg/mL and developed with ECL. (**a**) LPS (8 or 72 h) or saline treatment (8 h) (*n* = 3 for all groups). (**b**) Kainate (24 or 72 h) and saline groups (24 h) (*n* = 3 for all groups). (**c**) ME7 (13, 18, and 21 weeks) and NBH (21 weeks) (*n* = 4 for each group: one representative gel shown.).

Hippocampal homogenates were prepared at 8 h and 3 days after intracerebral injection with LPS and from animals 8 h after saline injection. Further homogenates were prepared from kainate-injected hippocampi 24 and 72 h after challenge or 24 h after saline injection. Homogenates from NBH animals (21 weeks) and ME7 animals (13, 18, and 21 weeks) were also prepared. These times are justified in the Experimental section. Homogenates were analyzed by PAGE and Western blotting for uPAR protein levels using an anti-mouse polyclonal antibody, and the results are summarized in Figure 2a. Because of its marked glycosylation, previously observed in mice (Cunningham et al.,2003b) and humans (Roldan et al.,^1990^), uPAR runs as a smear at between 45 and 60 kDa. LPS induced a marked expression of uPAR protein at 8 h postinjection and a dramatic increase by 3 days posttreatment (Fig. 2a). A further band at ∼35 kDa may represent the unglycosylated form of the protein (Roldan et al.,^1990^). Further data (Supp. Info. Fig. 2) show that the pattern of uPAR expression/glycosylation is not yet altered 2 h post-LPS, but is altered at 6 h and markedly so at 24 h. Immunoblots with mouse anti-β-actin and with goat anti-rabbit IgG on the same membrane verified that all lanes show approximately equal loading and that the observed bands are a consequence of binding of the rabbit antimurine uPAR antibody. uPAR was expressed at very low levels in saline-treated animals. Kainate-injected animals also showed clear expression of uPAR at 24 h postinjection with respect to saline-treated controls (Fig. 2b). This expression appeared to decrease by 3 days. ME7 animals showed a time-dependent increase in uPAR expression, with marked expression by 13 weeks and continued increases at 18 and 21 weeks, respectively (Fig. 2c). NBH controls showed no evidence of uPAR expression in the brain.

### Confirmation of both tPA and uPA Expression and Activity in the CNS

Because mRNA expression analysis indicated that both uPA and tPA activities may be present in some of the innate inflammatory responses under study, we examined this possibility further using both gel zymography and soluble assays in the presence of selective inhibitors. In these experiments, brain homogenates from progressive stages of prion disease were analyzed.

**Fig. 3:**
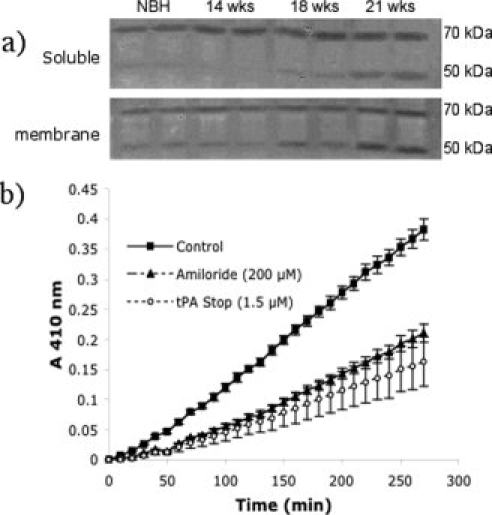
Zymographic and 96-well soluble assay of plasmin activity in ME7 animals. (**a**) Plasmin activity was assessed in both soluble and membrane fractions by in-gel zymography. The activity of both tPA and uPA are visible on the gel at MWs of ∼67 and 50 kDa, respectively (pro forms). Samples are in duplicate and one representative gel of two performed (*n* = 4) is shown. Images have been captured by digital camera and reversed to increase the clarity of the banding pattern. (**b**) Plasmin activity was assessed by 96 well assay using ChromazymPL as substrate. Data represent the mean ± SEM for *n* = 4 in each experimental group. Selective inhibition of plasmin activity at 18 weeks postinoculation with ME7 prion disease using selective inhibitors of tPA (tPA stop, 1.5 μm) and uPA (amiloride, 0.2 mM) revealed the presence of both activities in this membrane fraction.

The gel zymography method separates tPA and uPA activities on the basis of size, with tPA running at a molecular weight of ∼67 kDa and uPA, in its pro form, at ∼50 kDa. Figure 3a shows that uPA protein increases in a time-dependent manner during the course of prion disease, consistent with the mRNA data. Under the same conditions, tPA protein expression is constitutively high with less marked increases observed with disease progression. In this case, the majority of tPA is associated with the soluble fraction. Figure 3b illustrates that both uPA and tPA activities are present in membrane fractions of the ME7 brain, with comparable fractions of total plasminogen activation being inhibited by selective tPA and uPA inhibitors [tPA stop (1.5 μm) and amiloride (0.2 mM) respectively]. These inhibitors may also affect the activity of plasminogen activators even when receptor-bound, which may be significant given the ability of the plasminogen activators to act as soluble of receptor-bound forms.

### Plasminogen Activation During Distinct CNS Pathologies

**Fig. 4:**
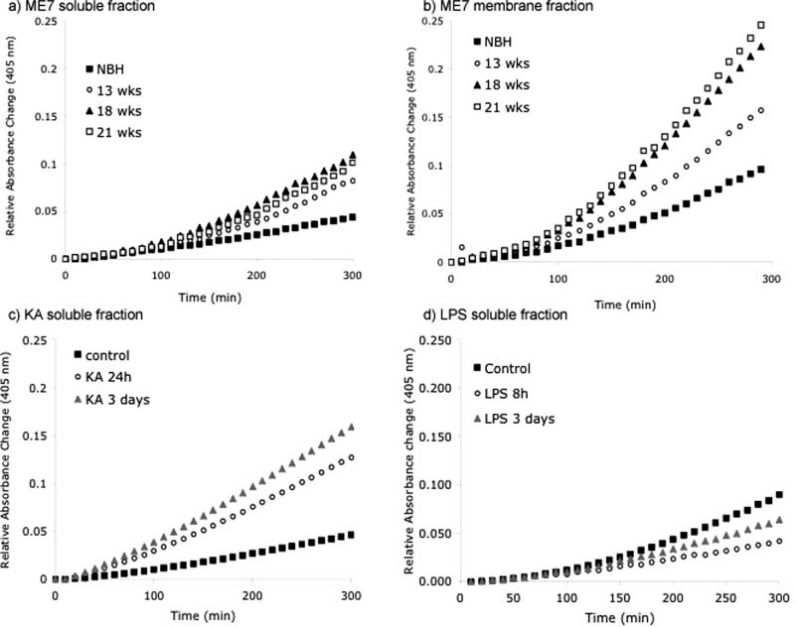
Total plasmin activity in soluble and/or membrane fractions of homogenates of hippocampal tissue from animals injected with NBH, ME7, LPS, KA, or saline. (**a**) Soluble fractions of NBH and ME7 (13, 18, and 21 weeks). (**b**) Membrane fractions of NBH and ME7 (13, 18, and 21 weeks). (**c**) Soluble fractions of KA (24 and 72 h) and saline-injected (24 h). (**d**) Soluble fractions of LPS (8 and 72 h) and saline-injected animals (8 h). Data represent the mean for *n* = 4 in each experimental group. Error bars have been omitted for clarity, owing to the frequency of sampling.

Gel zymography analysis showed the presence of both uPA and tPA in the brain and associated increases during the progression of prion disease. However, zymography cannot assess the activities of these enzymes in solution, because inhibitors such as PAI-1 may remain bound on the nonreducing gel. To assess the effects of the three distinct CNS insults on total plasminogen activation, activity was measured in total brain homogenates as described earlier (those used for zymography). Both soluble and membrane-associated homogenate fractions were analyzed in each case. An increase in total plasminogen activation with progression of prion disease is apparent in both soluble and membrane fractions (Fig. 4a,b, respectively). This increase is more apparent, and clearly time-dependent, in PA associated with the membrane fraction. Similar total PA increases are evident after kainate challenge (Fig. 4c). However, total PA activity decreases after LPS challenge (Fig. 4d). Only soluble fractions are shown for kainate and LPS challenges as the trend is similar for membrane-associated fractions in both cases.

### Cellular Localization of uPAR in the CNS

**Fig. 5:**
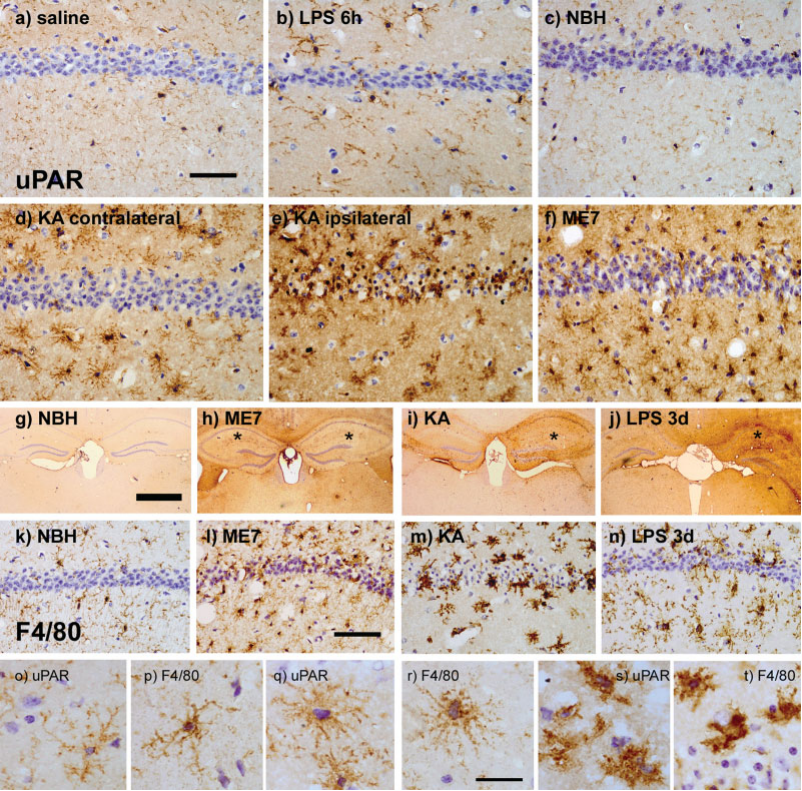
Cellular localization of uPAR expression. PLP-fixed brain sections were immunostained for uPAR (**a–j**) to identify the cellular source of uPAR in the brain after various insults and for F4/80 (**k–n**) to confirm morphology and location of macrophages/microglia following these insults. Low-level staining of highly ramified microglial processes can be observed in control animals; (a) saline (8 h) and (c) NBH (18 weeks). Increased expression is evident in (b) LPS (8 h) tissue and (d) contralateral to KA challenge, though the morphology remains quite ramified. Marked expression of uPAR is evident in amoeboid microglia ipsilateral to KA challenge (e) and in condensed microglia in ME7-treated animals (f). In addition to cellular staining, uPAR appears to be released into the parenchyma after ME7 (h), KA (i), and LPS (j) challenges. The site of injection is indicated by *. F4/80 staining reveals constitutive labelling of ramified microglial cells in NBH controls (k). The levels of F4/80 and cell morphology are altered in ME7 (l), KA (m), and LPS 3 days (n). uPAR and F4/80 are also shown at 100× magnification to illustrate the similar morphologies of the cells positively labeled for these antigens (**o–t**). Both markers display the ramified (saline: o, p), hyper-ramified (kainate contralateral: q, r) and activated/amoeboid (kainate ipsilateral: s, t). Scale bars = 70 μm (a–f), 1 mm (g–j), 50 μm (k–n), and 10 μm (o–t).

Immunolabeling revealed that uPAR is expressed in cells with typical, classical microglial morphologies (Streit et al.,^1999^) in all three conditions and also in infiltrating macrophages after LPS challenge. The staining levels are very low in saline-injected (Fig. 5a) and NBH-injected control animals (Fig. 5c), but there was nonetheless, labeling of the highly ramified processes of microglial cells in their normal quiescent state (5 o,p). The staining was clearly increased in animals 8-h post-LPS challenge, and this staining revealed microglial cells maintaining a ramified morphology (Fig. 5b). Animals examined 3 days after LPS challenge showed evidence of diffuse extracellular uPAR staining, apparently throughout the parenchyma, consistent with cleavage of GPI-linked uPAR to increase suPAR levels (Fig. 5j). Cleavage and shedding of uPAR is well documented in monocytes (Sidenius et al.,^2000^). In the same manner, uPAR was upregulated 3 days post-KA challenge on microglial cells with shortened processes in the vicinity of the dying neurons of the CA1 (Fig. 5e,i,s,t). Furthermore, the contralateral side to KA injection also showed hyper-ramified microglial staining with uPAR expression (Fig. 5d,q,r). ME7 animals also showed clear evidence of microglial uPAR staining (Fig. 5f) with microglia showing upregulation of uPAR throughout the hippocampus and dorsal thalamus, as has been shown previously for other microglial markers in this disease model (Betmouni et al.,^1996^). Similar to LPS and KA challenges, ME7 animals also showed evidence of diffuse uPAR staining in the parenchyma that was not associated with particular cells (Fig. 5h) and was increased relative to the very low-parenchymal labeling in NBH-treated animals (Fig. 5c,g).

We also stained for F4/80 after the various challenges used in this study to confirm the morphology and location of macrophages/microglia following these insults. F4/80 immunostaining revealed constitutive labeling of ramified microglial cells in NBH and saline-injected animals (Fig. 5k,p), and this morphology was shared by uPAR-positive cells (Fig. 5o). The levels of expression of the F4/80 antigen and the morphology of macrophages/microglia are altered in LPS, KA, and ME7-injected animals (Fig. 5n,m,l, respectively). This is most obvious ipsilateral to KA challenge, after which the microglial processes become shorter and condensed, and these cells invade the degenerating CA1 neuronal layer (Fig. 5m). This classical activated/amoeboid morphology is clearly shown by both uPAR and F4/80 (Fig. 5s,t). Significantly, the hyper-ramified morphology typical of “alert” microglia was also observed on the contralateral side to the kainate challenge and also is displayed by both markers (Fig. 5q,r). Thus, F4/80-staining parallels the cellular staining observed with the anti-uPAR antibody in its morphology and localization after the different challenges. Significantly, uPAR expression is present in resident microglial cells before any infiltration of cells from the bloodstream has taken place (Andersson et al.,^1992^).

## DISCUSSION

We have shown here that uPAR is expressed by resident microglial cells of the murine brain and that it is significantly upregulated in the brain in response to a number of challenges that provoke an innate immune response. At peak expression, there is considerable shedding of this GPI-linked protein from the cell surface into the brain parenchyma. This is the first clear demonstration, *in vivo*, that resident microglia as well as infiltrating macrophages expresses this protein. In addition, we have shown expression and activity of uPA in the murine brain, particularly during the progression of chronic neurodegeneration caused by the ME7 strain of murine prion disease. There are interesting dissociations between the inductions of uPA and uPAR among the various challenges studied here. Both LPS and kainate-induced neurodegeneration led to very marked inductions of uPAR with minimal effect on uPA expression, whereas chronic neurodegeneration induces uPA and uPAR transcriptional increases of similar magnitude (14–20-fold) perhaps indicating divergent roles of protease activity in these models.

### uPAR and uPA Expression in the Brain

Monocytes/macrophages are known to express uPAR (Min et al.,^1992^; Vassalli et al.,^1992^), and levels of expression are reported to be elevated during endotoxemia and by various cytokines including IL-1β, TNF-α, and TGFβ1 (Dekkers et al.,^2000^; Yue et al.,^2004^). The expression of uPAR and its site-directed focusing of uPA activity play a central role in cell migration and infiltration to sites of inflammation (Gyetko et al.,^2000^,^2004^). Low-level expression of uPAR has been described in microglial cells *in vitro*, and this expression was increased upon LPS stimulation; however, uPAR could not be detected in microglia immediately *ex vivo* by these authors (Washington et al.,^1996^). There have been reports of brain macrophage/microglial cell surface uPAR expression in HIV dementia, multiple sclerosis, cerebral malaria, traumatic brain injury, CJD, and Alzheimer's disease (Beschorner et al.,^2000^; Cinque etal.,^2004^; Deininger et al.,^2002^; Fauser et al.,^2000^; Gveric et al.,^2001^; Sidenius et al.,^2004^; Walker et al.,^2002^). Some of these studies show either double staining with macrophage markers or morphology suggestive of microglial cells. However, most of these studies describe conditions in which macrophages have recently infiltrated the brain. Even in a chronic disease, such as Alzheimer's disease (Walker et al.,^2002^), in which uPAR positive staining is colocalized with CD68-positive macrophages, it is not clear whether these are resident or infiltrating cells, because bone marrow-derived infiltrating macrophages have been reported to be selectively targeted to amyloid plaques in animal models of Alzheimer's disease (Simard et al.,^2006^).

In this study, we demonstrate that uPAR is expressed in resident microglial cells in the normal brain and in both acute and chronic inflammation. Immunohistochemistry reveals that uPAR is expressed at very low levels in naïve and saline-treated mice but is significantly upregulated by LPS many hours before cell infiltration occurs, demonstrating that resident microglial cells upregulate this cell surface marker upon stimulation. In light of its role in cell migration, the expression of uPAR by infiltrating macrophages in the brain was readily rationalized. Although the need for local extracellular protease activity is not so obvious for resident cells, recent data demonstrate that microglia continually explore their local microenvironment, and these cells are well known to respond rapidly to transient neural activity suppression by stimulation with potassium chloride (cortical spreading depression: (Gehrmann et al.,^1993^; Jander et al.,^2001^) and to show activation in undamaged regions, distal to ischemic lesions (Schroeter et al.,^1999^). In this regard, it is interesting that in our studies even saline challenges produce measurable increases in uPAR gene expression when compared with naïve animals and that uPAR is robustly expressed in hyper-ramified microglia contralateral to kainate challenges, indicating that even minor disturbances to the brain parenchyma provoke transcriptional, translational, and perhaps structural changes in microglial cells. The interaction of uPA with uPAR may thus contribute to morphological changes and migration toward relatively proximal stimuli.

It is also possible that uPAR has roles in brain inflammation that are entirely dissociated from the focusing of uPA activity. We show here that uPAR is highly expressed after both kainate and LPS challenges, but that uPA is not significantly upregulated under the same conditions. There is now a large body of evidence to support multiple proteolysis-independent roles for uPAR in cell adhesion, migration, proliferation, and differentiation outside the CNS [reviewed in Blasi and Carmeliet (2002)]. Recent experiments suggest that uPAR has a uPA-independent role in ischemic damage (Nagai et al.,^2008^). A second, key-binding partner of uPAR is the extracellular matrix protein vitronectin (VN). Overexpression of uPAR in HEK-293 cells *in vitro* and subsequent uPAR-VN interaction was shown to induce profound changes in cell morphology, including cell spreading and rearrangement of the actin cytoskeleton and focal adhesion contacts (Madsen et al.,^2007^). This cell–matrix interaction could be postulated to mediate the morphological changes that are observed upon activation of resident microglia, leading to the less-ramified, more-condensed morphology after inflammatory challenges. In addition, VN has been shown to be present in diffuse and dense core plaques in Alzheimer's disease (Akiyama et al.,^1991^), and its interaction with uPAR may also influence microglial activation at the plaque periphery.

We also observed variation in uPAR glycosylation with different challenges. Levels of uPAR protein expression are most robustly induced after acute LPS challenge, and it is also this condition that leads to the highest level of uPAR glycosylation (Fig. 2a). In contrast, uPAR glycosylation is not as marked during the development of prion disease (Fig. 2c). Time-dependent changes in uPAR glycosylation levels have previously been observed during monocyte activation, and this has been shown to be correlated with protein–protein interactions important in mediating cell adhesion (Mahoney et al.,^2001^). Similar alterations in *N*-glycosylation patterns have recently been reported in a number of inflammatory conditions including sepsis, acute pancreatitis, and systemic lupus erythematosus (Gornik et al.,^2007^; Hashii et al.,^2008^), and the significance of different glycosylation patterns in the current data merits further study.

The high levels of uPAR expression observed in the current study, during CNS inflammation induced by three distinct challenges (chronic neurodegeneration, acute neurotoxicity, and acute non-neurotoxic LPS), can perhaps explain the frequently reported elevations of suPAR in the CSF of patients with HIV dementia and other CNS conditions (Cinque et al.,^2004^; Garcia-Monco et al.,^2002^; Sporer et al.,^2005^; Winkler et al.,^2002^). Conversely, neither stroke nor demyelinating diseases such as MS or Guillian–Barre syndrome resulted in elevated CSF suPAR despite comprising significant inflammatory components (Garcia-Monco et al.,^2002^). It seems likely that release of suPAR into the CSF is directly related to increased CNS inflammation as we observed considerable diffuse staining, that was not cell-associated, in the parenchyma in addition to microglial labeling. This would suggest that uPAR is unlikely to have specific diagnostic utility for any particular CNS condition but is likely to be a reliable indicator of current CNS inflammation. It would appear that many types of inflammation result in shedding of suPAR from the microglial cell surface in the brain; however, other factors, currently unknown, may determine uPAR's escape to the CSF. Likewise, findings that cell-surface levels of uPAR are controlled by endocytosis and recycling (Cortese et al.,^2008^) should be replicated in microglia, although this would be more easily addressed using *in vitro* approaches.

### uPA or tPA?

Tissue plasminogen activator (tPA) is constitutively active in the rodent brain (Sappino et al.,^1993^) and contributes to the neurotoxicity of kainic acid in rodent studies (Tsirka et al.,^1995^,^1997^) and, in this regard, our finding that tPA rather than uPA is upregulated after kainate challenge is consistent with the consensus view of kainate-induced microglial expression of neurotoxic tPA. Nonetheless, there is clear evidence that uPA rather than tPA is the key plasminogen activator in cerebral ischemia in mice, rats, and humans (Cinelli et al.,^2001^; Hosomi et al.,^2001^; Rosenberg et al.,^1996^), and it has recently been described as coexpressed with uPA in brains of HIV patients with opportunistic cerebral infections (Nebuloni et al.,^2008^). So, what governs whether either or both plasminogen activators are switched on during inflammation? It is well described that uPA is robustly upregulated by macrophage CSF-1 (Stacey et al.,^1995^), and we have found evidence for increased transcription of CSF-1 in the ME7 prion disease model (unpublished observations). In addition, both tPA and uPA have been described to be differentially regulated in multiple cell types by IL-1α/β, TNFα, TGFβ1, and prostaglandin E2 (Allan and Martin,^1995^; Falcone et al.,^1993^,^1995^; Gerritsen et al.,^1993^; So et al.,^1992^; Zhang et al.,^1996^). All these mediators are produced by intracerebral LPS challenges (Boche et al.,^2003^), but TGFβ1 and PGE2 would appear to dominate in ME7-induced prion disease (Cunningham et al.,^2002^; Minghetti et al.,^2000^). Clearly, the regulation of tPA and uPA in the brain requires considerably more investigation.

There have been a number of studies published describing a role for plasminogen activation in prion disease, but these studies have been focussed on tPA. Initial studies demonstrated a direct interaction between plasminogen and disease-associated PrP (Fischer et al.,^2000^; Maissen et al.,^2001^), and subsequent *in vitro* studies suggested that PrP^Sc^ specifically activated tPA without effect on uPA (Ellis et al.,^2002^; Epple et al.,^2004^). Despite this, there is evidence that tPA cannot degrade PrP^Sc^ (Xanthopoulos et al.,^2005^). The role of plasminogen and tPA in prion disease is further clouded by divergent results when plasminogen deficient animals (plg−/−) were inoculated with prion disease: one study reports a very slight protection of plg−/− animals (Salmona et al.,^2005^), while the other reports that plasminogen deficiency prompts earlier symptoms and death (Xanthopoulos et al.,^2005^). These studies are both compromised by the adverse phenotype of the plasminogen knockout mouse, which shows poor health, retarded growth, and shortly after inoculation, becomes runted, listless, and cachectic (Salmona et al.,^2005^). With such an adverse phenotype attributing symptoms and/or death to prion disease rather than gene deficiency become problematic, perhaps reflected in the very large variability observed in the studies of Xanthopoulos et al. (2005). Our current studies are suggestive of increases in both tPA and uPA, as shown by partial inhibition by both amiloride and tPA, and by the presence of both activities in zymographic analysis. In addition, the increased transcription of uPA is very prominent in this disease model given its very low constitutive expression. Whether uPA synthesis is a response to PrP^Sc^ deposition, has a role in microglial migration/phagocytosis in concert with uPAR, or affects some other aspect of neuroprotection/neurodegeneration remains unclear but certainly merits further study.

### The Influence of PAI-1

In models of Alzheimer's disease, both tPA and uPA have been reported to show increased expression (Tucker et al.,^2000^). However, tPA activity has been shown by *in situ* zymography to be decreased in AD models, and its activity is proposed to be controlled by the substantial increases in PAI-1 (Melchor et al.,^2003^). We also observed PAI-1 elevation in prion disease but despite its probable presence in the soluble assay, we still observe considerable increases in plasmin activation (Fig. 3a). This raises an interesting point about PAI-1 expression. In the current studies, we have observed clear increases in plasmin activity in both ME7- and KA-treated animals, but decreased activity in LPS-treated animals. LPS produced the most marked transcriptional increase in PAI-1, suggesting that PAI-1 expression may be the ultimate determinant of *in vivo* plasmin activity. IL-1β, TNF-α, and TGFβ1 have all been shown to induce PAI-1 robustly and to bring about decreased plasminogen activation (Faber-Elman et al.,^1995^), and it is well known that LPS i.c. challenges induce all these cytokines in the mouse brain (Boche et al.,^2003^). Because tPA contributes to neuronal death during excitotoxicity and uPA to ischemic and cytokine-induced neurotoxicity (Rosenberg et al.,^1996^; Thornton et al.,^2008^) suppression of these activities during LPS challenges may be one major reason why this robust inflammatory stimulus is not overtly neurotoxic. Likewise, the dominance of tPA/uPA over their inhibitors in prion disease may have a beneficial effect, in clearing PrP^Sc^, or a deleterious effect in causing bystander proteolytic damage. Furthermore, the balance of PAI-1 and tPA/uPA from a neuroanatomical point of view may be key in this regard. Preliminary *in situ* zymography data suggest that both plasminogen activator activities are present in the hippocampus (not shown), but considerably more work is required to clarify this. Nonetheless, specific targeting of uPA and/or PAI-1 may produce interesting effects in the ME7 model of prion disease, irrespective of previous manipulations of tPA.

## CONCLUSION

A number of clinical studies have shown that uPAR is elevated in the CSF of patients with a variety of CNS degenerative and/or inflammatory diseases. These clinical studies have not been matched by similar basic research efforts to understand the involvement of uPAR in these inflammatory situations. This study suggests that uPAR may be involved in a very wide variety of inflammatory and pathological situations in the brain and systematic studies of its role and that of uPA may contribute significantly to our understanding of many of these conditions and to a greater understanding of microglial biology.

## References

[b1] Akiyama H, Kawamata T, Dedhar S, McGeer PL (1991). Immunohistochemical localization of vitronectin, its receptor and β-3 integrin in Alzheimer brain tissue. J Neuroimmunol.

[b2] Allan EH, Martin TJ (1995). Prostaglandin E2 regulates production of plasminogen activator isoenzymes, urokinase receptor, and plasminogen activator inhibitor-1 in primary cultures of rat calvarial osteoblasts. J Cell Physiol.

[b3] Andersson PB, Perry VH, Gordon S (1991a). The CNS acute inflammatory response to excitotoxic neuronal cell death. Immunol Lett.

[b4] Andersson PB, Perry VH, Gordon S (1991b). The kinetics and morphological characteristics of the macrophage-microglial response to kainic acid-induced neuronal degeneration. Neuroscience.

[b5] Andersson PB, Perry VH, Gordon S (1992). The acute inflammatory response to lipopolysaccharide in CNS parenchyma differs from that in other body tissues. Neuroscience.

[b6] Beschorner R, Schluesener HJ, Nguyen TD, Magdolen V, Luther T, Pedal I, Mattern R, Meyermann R, Schwab JM (2000). Lesion-associated accumulation of uPAR/CD87- expressing infiltrating granulocytes, activated microglial cells/macrophages and upregulation by endothelial cells following TBI, FCI in humans. Neuropathol Appl Neurobiol.

[b7] Betmouni S, Perry VH, Gordon JL (1996). Evidence for an early inflammatory response in the central nervous system of mice with scrapie. Neuroscience.

[b8] Blasi F, Carmeliet P (2002). uPAR: A versatile signalling orchestrator. Nat Rev Mol Cell Biol.

[b9] Boche D, Cunningham C, Gauldie J, Perry VH (2003). Transforming growth factor-β 1-mediated neuroprotection against excitotoxic injury in vivo. J Cereb Blood Flow Metab.

[b10] Cacquevel M, Launay S, Castel H, Benchenane K, Cheenne S, Buee L, Moons L, Delacourte A, Carmeliet P, Vivien D (2007). Ageing and amyloid-β peptide deposition contribute to an impaired brain tissue plasminogen activator activity by different mechanisms. Neurobiol Dis.

[b11] Carmeliet P, Schoonjans L, Kieckens L, Ream B, Degen J, Bronson R, De Vos R, van den Oord JJ, Collen D, Mulligan RC (1994). Physiological consequences of loss of plasminogen activator gene function in mice. Nature.

[b12] Cinelli P, Madani R, Tsuzuki N, Vallet P, Arras M, Zhao CN, Osterwalder T, Rulicke T, Sonderegger P (2001). Neuroserpin, a neuroprotective factor in focal ischemic stroke. Mol Cell Neurosci.

[b13] Cinque P, Nebuloni M, Santovito ML, Price RW, Gisslen M, Hagberg L, Bestetti A, Vago G, Lazzarin A, Blasi F, Sidenus N (2004). The urokinase receptor is overexpressed in the AIDS dementia complex and other neurological manifestations. Ann Neurol.

[b14] Cortese K, Sahores M, Madsen CD, Tacchetti C, Blasi F (2008). Clathrin and LRP-1-independent constitutive endocytosis and recycling of uPAR. PLoS ONE.

[b15] Cunningham C, Deacon R, Wells H, Boche D, Waters S, Diniz CP, Scott H, Rawlins JN, Perry VH (2003a). Synaptic changes characterize early behavioural changes in the ME7 model of murine prion disease. Eur J Neurosci.

[b16] Cunningham O, Andolfo A, Santovito ML, Iuzzolino L, Blasi F, Sidenius N (2003b). Dimerization controls the lipid raft partitioning of uPAR/CD87 and regulates its biological functions. EMBO J.

[b17] Cunningham C, Boche D, Perry VH (2002). Transforming growth factor β1, the dominant cytokine in murine prion disease: Influence on inflammatory cytokine synthesis and alteration of vascular extracellular matrix. Neuropathol Appl Neurobiol.

[b18] Cunningham C, Wilcockson DC, Boche D, Perry VH (2005). Comparison of inflammatory and acute-phase responses in the brain and peripheral organs of the ME7 model of prion disease. J Virol.

[b19] Deininger MH, Trautmann K, Magdolen V, Luther T, Schluesener HJ, Meyermann R (2002). Cortical neurons of Creutzfeldt-Jakob disease patients express the urokinase-type plasminogen activator receptor. Neurosci Lett.

[b20] Dekkers PE, ten Hove T, te Velde AA, van Deventer SJ, van Der Poll T (2000). Upregulation of monocyte urokinase plasminogen activator receptor during human endotoxemia. Infect Immun.

[b21] East E, Baker D, Pryce G, Lijnen HR, Cuzner ML, Gveric D (2005). A role for the plasminogen activator system in inflammation and neurodegeneration in the central nervous system during experimental allergic encephalomyelitis. Am J Pathol.

[b22] Ellis V, Daniels M, Misra R, Brown DR (2002). Plasminogen activation is stimulated by prion protein and regulated in a copper-dependent manner. Biochemistry.

[b23] Epple G, Langfeld K, Baier M, Holzhutter HG, Schleuning WD, Kottgen E, Gessner R, Praus M (2004). Both lysine-clusters of the NH2-terminal prion-protein fragment PrP23-110 are essential for t-PA mediated plasminogen activation. Thromb Haemost.

[b24] Faber-Elman A, Miskin R, Schwartz M (1995). Components of the plasminogen activator system in astrocytes are modulated by tumor necrosis factor-α and interleukin-1 β through similar signal transduction pathways. J Neurochem.

[b25] Falcone DJ, McCaffrey TA, Haimovitz-Friedman A, Garcia M (1993). Transforming growth factor-β 1 stimulates macrophage urokinase expression and release of matrix-bound basic fibroblast growth factor. J Cell Physiol.

[b26] Falcone DJ, McCaffrey TA, Mathew J, McAdam K, Borth W (1995). THP-1 macrophage membrane-bound plasmin activity is up-regulated by transforming growth factor-β 1 via increased expression of urokinase and the urokinase receptor. J Cell Physiol.

[b27] Fauser S, Deininger MH, Kremsner PG, Magdolen V, Luther T, Meyermann R, Schluesener HJ (2000). Lesion associated expression of urokinase-type plasminogen activator receptor (uPAR, CD87) in human cerebral malaria. J Neuroimmunol.

[b28] Fischer MB, Roeckl C, Parizek P, Schwarz HP, Aguzzi A (2000). Binding of disease-associated prion protein to plasminogen. Nature.

[b29] Gabay C, Kushner I (1999). Acute-phase proteins and other systemic responses to inflammation. N Engl J Med.

[b30] Garcia-Monco JC, Coleman JL, Benach JL (2002). Soluble urokinase receptor (uPAR, CD 87) is present in serum and cerebrospinal fluid in patients with neurologic diseases. J Neuroimmunol.

[b31] Gehrmann J, Mies G, Bonnekoh P, Banati R, Iijima T, Kreutzberg GW, Hossmann KA (1993). Microglial reaction in the rat cerebral cortex induced by cortical spreading depression. Brain Pathol.

[b32] Gerritsen ME, Niedbala MJ, Szczepanski A, Carley WW (1993). Cytokine activation of human macro- and microvessel-derived endothelial cells. Blood Cells.

[b33] Gornik O, Royle L, Harvey DJ, Radcliffe CM, Saldova R, Dwek RA, Rudd P, Lauc G (2007). Changes of serum glycans during sepsis and acute pancreatitis. Glycobiology.

[b34] Gveric D, Hanemaaijer R, Newcombe J, van Lent NA, Sier CF, Cuzner ML (2001). Plasminogen activators in multiple sclerosis lesions: Implications for the inflammatory response and axonal damage. Brain.

[b35] Gyetko MR, Aizenberg D, Mayo-Bond L (2004). Urokinase-deficient and urokinase receptor-deficient mice have impaired neutrophil antimicrobial activation in vitro. J Leukoc Biol.

[b36] Gyetko MR, Sud S, Kendall T, Fuller JA, Newstead MW, Standiford TJ (2000). Urokinase receptor-deficient mice have impaired neutrophil recruitment in response to pulmonary *Pseudomonas aeruginosa* infection. J Immunol.

[b37] Harvey R, Chintala SK (2007). Inhibition of plasminogen activators attenuates the death of differentiated retinal ganglion cells and stabilizes their neurite network in vitro. Invest Ophthalmol Vis Sci.

[b38] Hashii N, Kawasaki N, Itoh S, Nakajima Y, Kawanishi T, Yamaguchi T (2008). Alteration of *N*-glycosylation in the kidney in a mouse model of systemic lupus erythematosus: Relative quantification of *N*-glycans using an isotope-tagging method. Immunology.

[b39] Hosomi N, Lucero J, Heo JH, Koziol JA, Copeland BR, del Zoppo GJ (2001). Rapid differential endogenous plasminogen activator expression after acute middle cerebral artery occlusion. Stroke.

[b40] Jander S, Schroeter M, Peters O, Witte OW, Stoll G (2001). Cortical spreading depression induces proinflammatory cytokine gene expression in the rat brain. J Cereb Blood Flow Metab.

[b41] Lund LR, Romer J, Ronne E, Ellis V, Blasi F, Dano K (1991). Urokinase-receptor biosynthesis, mRNA level and gene transcription are increased by transforming growth factor β 1 in human A549 lung carcinoma cells. EMBO J.

[b42] Madsen CD, Ferraris GM, Andolfo A, Cunningham O, Sidenius N (2007). uPAR-induced cell adhesion and migration: Vitronectin provides the key. J Cell Biol.

[b43] Mahoney TS, Weyrich AS, Dixon DA, McIntyre T, Prescott SM, Zimmerman GA (2001). Cell adhesion regulates gene expression at translational checkpoints in human myeloid leukocytes. Proc Natl Acad Sci USA.

[b44] Maissen M, Roeckl C, Glatzel M, Goldmann W, Aguzzi A (2001). Plasminogen binds to disease-associated prion protein of multiple species. Lancet.

[b45] Melchor JP, Pawlak R, Strickland S (2003). The tissue plasminogen activator-plasminogen proteolytic cascade accelerates amyloid-beta (Aβ) degradation and inhibits Aβ-induced neurodegeneration. J Neurosci.

[b46] Melchor JP, Strickland S (2005). Tissue plasminogen activator in central nervous system physiology and pathology. Thromb Haemost.

[b47] Min HY, Semnani R, Mizukami IF, Watt K, Todd RF, Liu DY (1992). cDNA for Mo3, a monocyte activation antigen, encodes the human receptor for urokinase plasminogen activator. J Immunol.

[b48] Minghetti L, Greco A, Cardone F, Puopolo M, Ladogana A, Almonti S, Cunningham C, Perry VH, Pocchiari M, Levi G (2000). Increased brain synthesis of prostaglandin E2 and F2-isoprostane in human and experimental transmissible spongiform encephalopathies. J Neuropathol Exp Neurol.

[b49] Nagai N, Okada K, Kawao N, Ishida C, Ueshima S, Collen D, Matsuo O (2008). Urokinase-type plasminogen activator receptor (uPAR) augments brain damage in a murine model of ischemic stroke. Neurosci Lett.

[b50] Nebuloni M, Cinque P, Sidenius N, Ferri A, Lauri E, Omodeo-Zorini E, Zerbi P, Vago L (2008). Expression of the urokinase plasminogen activator receptor (uPAR) and its ligand (uPA) in brain tissues of human immunodeficiency virus patients with opportunistic cerebral diseases. J Neurovirol.

[b51] Roldan AL, Cubellis MV, Masucci MT, Behrendt N, Lund LR, Dano K, Appella E, Blasi F (1990). Cloning and expression of the receptor for human urokinase plasminogen activator, a central molecule in cell surface, plasmin dependent proteolysis. EMBO J.

[b52] Rosenberg GA, Navratil M, Barone F, Feuerstein G (1996). Proteolytic cascade enzymes increase in focal cerebral ischemia in rat. J Cereb Blood Flow Metab.

[b53] Salmona M, Capobianco R, Colombo L, De Luigi A, Rossi G, Mangieri M, Giaccone G, Quaglio E, Chiesa R, Donati MB, Tagliavini F, Forloni G (2005). Role of plasminogen in propagation of scrapie. J Virol.

[b54] Sappino AP, Madani R, Huarte J, Belin D, Kiss JZ, Wohlwend A, Vassalli JD (1993). Extracellular proteolysis in the adult murine brain. J Clin Invest.

[b55] Schroeter M, Jander S, Witte OW, Stoll G (1999). Heterogeneity of the microglial response in photochemically induced focal ischemia of the rat cerebral cortex. Neuroscience.

[b56] Sidenius N, Nebuloni M, Sala S, Zerbi P, Price RW, Gisslen M, Hagberg L, Vago L, Lazzarin A, Blasi F, Cinque P (2004). Expression of the urokinase plasminogen activator and its receptor in HIV-1-associated central nervous system disease. J Neuroimmunol.

[b57] Sidenius N, Sier CF, Blasi F (2000). Shedding and cleavage of the urokinase receptor (uPAR): Identification and characterisation of uPAR fragments in vitro and in vivo. FEBS Lett.

[b58] Simard AR, Soulet D, Gowing G, Julien JP, Rivest S (2006). Bone marrow-derived microglia play a critical role in restricting senile plaque formation in Alzheimer's disease. Neuron.

[b59] So T, Ito A, Sato T, Mori Y, Hirakawa S (1992). Tumor necrosis factor-α stimulates the biosynthesis of matrix metalloproteinases and plasminogen activator in cultured human chorionic cells. Biol Reprod.

[b60] Sporer B, Koedel U, Popp B, Paul R, Pfister HW (2005). Evaluation of cerebrospinal fluid uPA, PAI-1, and soluble uPAR levels in HIV-infected patients. J Neuroimmunol.

[b61] Stacey KJ, Fowles LF, Colman MS, Ostrowski MC, Hume DA (1995). Regulation of urokinase-type plasminogen activator gene transcription by macrophage colony-stimulating factor. Mol Cell Biol.

[b62] Streit WJ, Walter SA, Pennell NA (1999). Reactive microgliosis. Prog Neurobiol.

[b63] Thornton P, Pinteaux E, Allan SM, Rothwell NJ (2008). Matrix metalloproteinase-9 and urokinase plasminogen activator mediate interleukin-1-induced neurotoxicity. Mol Cell Neurosci.

[b64] Tjwa M, Sidenius N, Moura R, Jansen S, Theunissen K, Andolfo A, De Mol M, Dewerchin M, Moons L, Blasi F, Verfaillie C, Carmeliet P (2009). Membrane-anchored uPAR regulates the proliferation, marrow pool size, engraftment, and mobilization of mouse hematopoietic stem/progenitor cells. J Clin Invest.

[b65] Tsirka SE, Bugge TH, Degen JL, Strickland S (1997). Neuronal death in the central nervous system demonstrates a non-fibrin substrate for plasmin. Proc Natl Acad Sci USA.

[b66] Tsirka SE, Gualandris A, Amaral DG, Strickland S (1995). Excitotoxin-induced neuronal degeneration and seizure are mediated by tissue plasminogen activator. Nature.

[b67] Tucker HM, Kihiko M, Caldwell JN, Wright S, Kawarabayashi T, Price D, Walker D, Scheff S, McGillis JP, Rydel RE, Estus S (2000). The plasmin system is induced by and degrades amyloid-β aggregates. JNeurosci.

[b68] Vassalli JD, Wohlwend A, Belin D (1992). Urokinase-catalyzed plasminogen activation at the monocyte/macrophage cell surface: A localized and regulated proteolytic system. Curr Top Microbiol Immunol.

[b69] Walker DG, Lue LF, Beach TG (2002). Increased expression of the urokinase plasminogen-activator receptor in amyloid β peptide-treated human brain microglia and in AD brains. Brain Res.

[b70] Walsh DT, Betmouni S, Perry VH (2001). Absence of detectable IL-1β production in murine prion disease: A model of chronic neurodegeneration. J Neuropathol Exp Neurol.

[b71] Washington RA, Becher B, Balabanov R, Antel J, Dore-Duffy P (1996). Expression of the activation marker urokinase plasminogen-activator receptor in cultured human central nervous system microglia. J Neurosci Res.

[b72] Wilcockson DC, Campbell SJ, Anthony DC, Perry VH (2002). The systemic and local acute phase response following acute brain injury. J Cereb Blood Flow Metab.

[b73] Winkler F, Kastenbauer S, Koedel U, Pfister HW (2002). Role of the urokinase plasminogen activator system in patients with bacterial meningitis. Neurology.

[b74] Xanthopoulos K, Paspaltsis I, Apostolidou V, Petrakis S, Siao CJ, Kalpatsanidis A, Grigoriadis N, Tsaftaris A, Tsirka SE, Sklaviadis T (2005). Tissue plasminogen activator in brain tissues infected with transmissible spongiform encephalopathies. Neurobiol Dis.

[b75] Yue J, Sun B, Liu G, Mulder KM (2004). Requirement of TGF-β receptor-dependent activation of c-Jun N-terminal kinases (JNKs)/stress-activated protein kinases (Sapks) for TGF-β up-regulation of the urokinase-type plasminogen activator receptor. J Cell Physiol.

[b76] Zhang X, Shu MA, Ross HE, Kennedy TG (1996). Regulation of plasminogen activator in rat endometrial stromal cells: The role of prostaglandin E2. Biol Reprod.

