# Supplementary material for: Microglia and the urokinase plasminogen activator receptor/uPA system in innate brain inflammation
Source: Glia. 2009 May 20;57(16):1802–14. doi: 10.1002/glia.20892 (PMC2816357; doi:10.1002/glia.20892)
Supplement: Supplementary file 1 [file glia0057-1802-SD1.doc]

**Supporting Information.**

A number of controls have been included here to supplement information provided in the main text with respect to specificity of antibodies against uPAR.

The appropriate detection of murine uPAR expressed in Chinese Hamster Ovary cells, and the lack of non-specific bands in mock-transfected cells is shown in figure 1. These blots also show the broad range glycosylation described in previous publications on uPAR and replicated in the main manuscript. Thus the polyclonal anti-murine uPAR antibody detects murine uPAR. The secondary antibody used to recognize this rabbit primary antibody does not produce any positively stained bands when immunoblotting is carried out in the absence of the primary antibody (Figure 2). In addition, the same antibody produced cellular staining in the kidney of normal mice while no staining was present in uPAR^-/-^ mice (Tjwa et al., 2009; see rebuttal letter for images).

1 2 3 4 5


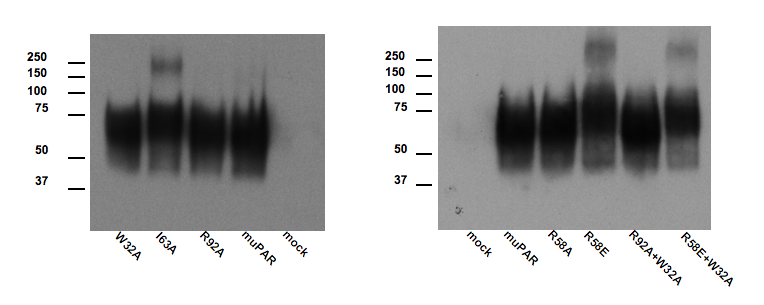


**Supporting Information Figure 1**. WB analysis, using affinity purified rabbit polyclonal anti-uPAR SI420, of CHO cells stably expressing murine uPAR. Chinese hamster ovary cells transfected with vector expressing uPAR (lane 4) or not (mock-transfected, lane 5).

The Western blots shown in figure 2 of the paper did not include loading controls since we judged that the very marked qualitative changes in certain treatment groups could not be explained by slight variability of total quantity of protein loaded. Further SDS PAGE and western blots (Figure 2) verify this. Animals treated with 1 µg LPS i.c. and euthanised at 2 hours, 6 hours or 24 hours show patterns of uPAR expression and glycosylation comparable to those shown in figure 2a of the main paper. The increased level and distinctive broad range glycosylation of uPAR is evident at 6 hours and very marked at 24 hours, while it is absent at 2 hours and in saline-treated controls. The intensity of bands immunoblotted for β-actin does not vary significantly on the same blots (middle panel). Controls showing the same membrane incubated in the presence of goat anti-rabbit IgG secondary antibody, but in the absence of the rabbit anti-murine uPAR are also included (figure 2 bottom panel).


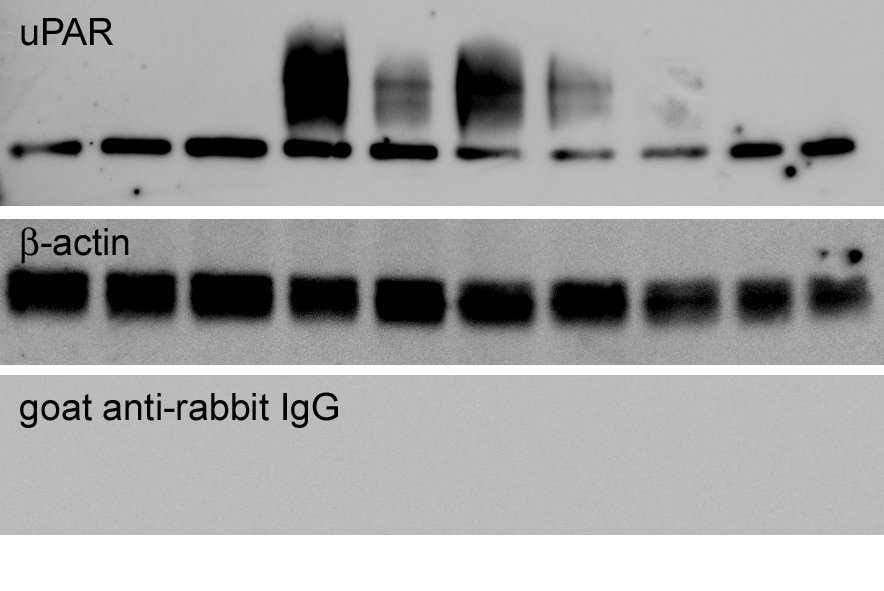


1 2 3 4 5 6 7 8 9 10

**Supporting Information Figure 2**. Western blots for uPAR (top) and β-actin (middle) and goat anti-rabbit IgG secondary antibody (bottom) performed on hippocampal homogenates of animals treated intra-cerebrally with LPS or saline. Lanes are as follows: 1-3 LPS 2h, 4-6 LPS 24 h, 7 & 8, LPS 6h, 9 & 10 saline.

The monoclonal anti murine uPAR antibody used in this study was recognized by a horse anti-mouse IgG. This secondary antibody when applied in the absence of the primary antibody did not produce any staining in the parenchyma of murine hippocampus, even when prion-infected, as shown in figure 3 below.


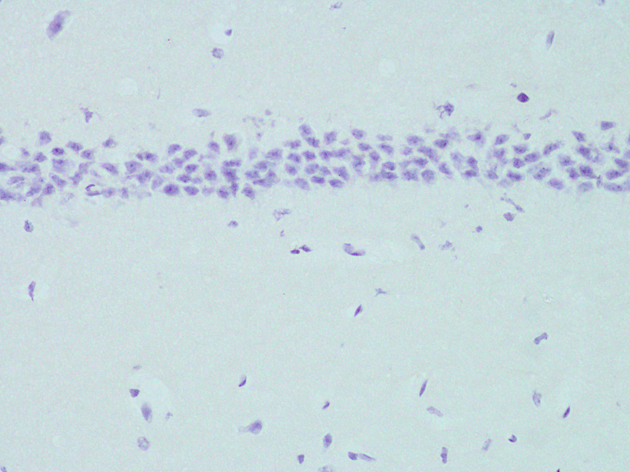


**Supporting Information Figure 3**. Negative control: Murine hippocampus stained with horse anti-mouse IgG.
